# Word Generalization by a Dog (*Canis familiaris*): Is Shape Important?

**DOI:** 10.1371/journal.pone.0049382

**Published:** 2012-11-21

**Authors:** Emile van der Zee, Helen Zulch, Daniel Mills

**Affiliations:** 1 School of Psychology, Brayford Campus, University of Lincoln, Lincoln, United Kingdom; 2 School of Life Sciences, Riseholme Campus, University of Lincoln, Lincoln, United Kingdom; University of Arizona, United States of America

## Abstract

We investigated the presence of a key feature of human word comprehension in a five year old Border Collie: the generalization of a word referring to an object to other objects of the same shape, also known as shape bias. Our first experiment confirmed a solid history of word learning in the dog, thus making it possible for certain object features to have become central in his word comprehension. Using an experimental paradigm originally employed to establish shape bias in children and human adults we taught the dog arbitrary object names (e.g. *dax*) for novel objects. Two experiments showed that when briefly familiarized with word-object mappings the dog did not generalize object names to object shape but to object size. A fourth experiment showed that when familiarized with a word-object mapping for a longer period of time the dog tended to generalize the word to objects with the same texture. These results show that the dog tested did not display human-like word comprehension, but word generalization and word reference development of a qualitatively different nature compared to humans. We conclude that a shape bias for word generalization in humans is due to the distinct evolutionary history of the human sensory system for object identification and that more research is necessary to confirm qualitative differences in word generalization between humans and dogs.

## Introduction

The question of whether dogs share any linguistic abilities with humans is the focus of much recent research [1]–[4]. It is an important question, since insights into the linguistic abilities of other species may give us insight into the evolutionary processes that led to human language [5]–[9]. For example, if dogs generalize comprehended words to the same objects as humans do this might be an indication that the mammalian brain is primed for word comprehension in a particular fashion. This paper considers an experimental investigation of word comprehension in a domestic dog, compares it with word comprehension in humans, and considers what this comparison may tell us about the evolution of word comprehension in humans.

It is now clear that dogs can treat words as verbal referents [1]–[4]. Rico, a nine year old Border Collie, was reported to know the meaning of 200 words, as demonstrated by his ability to fetch objects upon hearing their name [1]. The authors compared Rico's rate and manner of word learning to word learning processes in young children, and also claimed that Rico's memory of object words (such as *ball*) was comparable to that of a three year old toddler. Chaser, a three year old Border Collie, was reported to know the names of more than 1000 objects, thus showing that from a quantitative perspective there is no difference between the number of words that can be learned by the domestic dog and very young children [2]. Chaser also showed the ability to associate a word (for example, *toy*) with categories of objects as can children and human adults. However, despite these apparently impressive feats of verbal referencing, several arguments have been put forward to dispute claims that word knowledge in the domestic dog is of the same quality as word knowledge in humans. For example, it has been questioned [9], [10] that a dog is able to retrieve named objects in contexts or with instructors other than those involved in the initial object name learning process, that dogs can associate objects with object words if the latter are embedded in strings of other words, if different measures are used (for example, pointing instead of retrieving), or if objects are used that are not easily retrievable (for example, objects that are too large to be picked up). Recent work shows that a mongrel can reliably retrieve objects or point to them by using its nose on the basis of two-word string commands (for example, *stick point*) [3], and a Yorkshire Terrier has been shown to reliably retrieve objects upon receiving instructions that are unrelated to the voice and accent with which it was trained [4]. Although these findings seem to suggest qualitative similarity in word comprehension in the dog and in humans, the presence of a well-established feature characterizing the quality of human word comprehension has so far not been investigated in the dog: a bias to link the meaning of words referring to objects to object shape [12]–[24]. We investigated the presence of this so-called shape bias in the domestic dog.

Landau, Smith and Jones (LSJ) [12] originally showed that when two and three year old children or adults learnt to connect a new object name with a new object, they generalized the meaning of the new name to objects that are similar in shape, but not to objects that are similar in size or texture. For example, LSJ showed children and adults a U-shaped solid object (an object it could be assumed they had never seen before) calling it a *dax* (a name they had never heard before) and with the DAX object in sight asked them to select one of two objects they would also call a *dax*. The two objects they were asked to choose from could differ in shape, size or texture. In a significant number of cases children and adults choose objects that only differed in size or texture but not in shape, thus showing they tended to associate the name of an object with its shape, but not with its size or texture. In LSJ's experiments objects and names were used with which the participants had no previous experience, so that any learnt associations with the objects or names could not influence the results. The shape bias as detected by LSJ is widely acknowledged as playing a role in extending a learnt object name to other objects in both artificial and spontaneous word learning in children and adults (for example, in extending the word *cup* to many instances of cups), although the causes of the shape bias have been the subject of much debate [13]–[24]. In our paper we focus on whether word reference in the domestic dog is also determined by a shape bias, or whether object size or texture play a role in generalizing the meaning of comprehended words to new objects, as originally investigated by LSJ.

Would it be possible for a dog to rely on object shape in word comprehension in principle? Object discrimination tasks with dogs show that the domestic dog is able to use object shape but also object size to identify objects [25], [26]. This means that dogs can use shape information or size information to distinguish between objects when learning names for objects. However, employing the experimental set-up used by LSJ [12] we investigated which object features a dog will *spontaneously* select for named objects: shape – as in humans – or object size or texture. By focusing on the object features used in LSJ's experiments it is possible to directly compare object features in word generalization between humans and the dog.

Our test participant was a five year old Border Collie (Gable) with a history of word learning. Working with a dog with a history of word learning made it more likely that new words could be learnt, and that certain object features had become central in word learning [17]. It was claimed by his owner that Gable knew at least 54 different words referring to different objects. In our first experiment we tested whether Gable indeed knew 54 different words for 54 different objects. The second and fourth experiments were partial replications of LSJ's classical shape bias study [12]. Experiment 2 studied the nature of Gable's word generalization after he had been familiarized with a new object-new name combination for approximately 10 minutes, whereas experiment 4 studied Gable's word generalization after he had been familiarized with a new object-new name combination for 39 days. We were interested in whether the referential properties of a word which had been known by Gable for a longer period would be stable over time, as the shape bias is in humans [12]. Experiment 3 investigated whether the size bias that Gable had demonstrated in experiment 2 was due to the nature of the stimuli used, or whether this bias generalized to other artificial shapes. After presenting our experimental results we consider whether it is possible to explain any object feature bias on the basis of the input that Gable had received in the three years of word learning prior to our testing.

## General Methods

### Ethics

This research was approved by the School of Life Sciences Ethics Committee at the University of Lincoln, UK. The participants in the video clips that are part of the supplementary materials have given written informed consent, as outlined in the PLOS consent form, to publication of themselves.

### Participant

All experiments were conducted with Gable, a 5 year old Border Collie. Gable's owner reported that she had used social reinforcement (praise or play) to teach Gable word-object combinations. New objects and new object names were introduced by holding a new object in view, sounding out the object name several times, and then giving Gable the opportunity to play with the object while the word was repeated again. Gable would then be asked to retrieve the new object upon hearing his name and one or two instances of the new word with a fetch command (for example, *Gable, x; get x*), and following retrieval Gable would place the object in a container. Next, the object would be placed among other objects for which Gable knew the name (between five and 15 objects), after which Gable was prompted to select the new object from the set. This procedure illustrates a word game that Gable had played for approximately three years preceding the experiments.

### Experiment 1: establishing Gable's word comprehension ability

The purpose of the first experiment was to establish whether Gable comprehended the 54 words he was claimed to know by his owner.

#### A) Methods

54 of Gable's toys were divided into 26 sets of 10 objects that were randomly selected from the total set. Gable was asked to retrieve two objects from each set. In two cases Gable was asked to retrieve one item from a set of 10 randomly chosen objects. Gable had breaks between every five sets of objects, and no more than 10 sets were presented on a single testing day.

**Figure 1:**
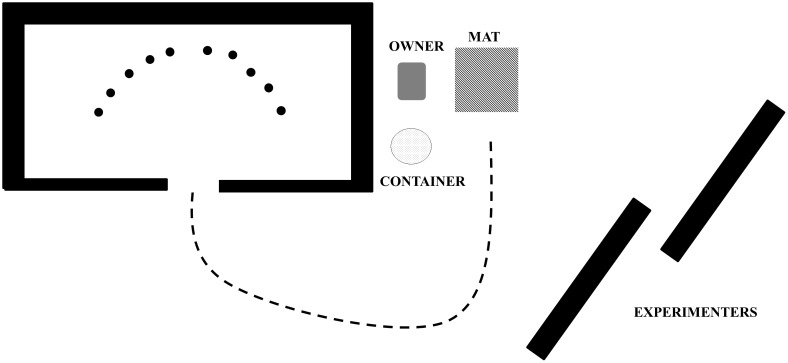
The lay-out of the area in which Gable was tested.

The testing area was separated from a larger room by portable barriers, and was approximately 2.5 m long and 5 m wide (see Figure 1). The barriers were positioned in such a way as to leave a central gap through which Gable could enter the testing area. All test items were presented inside the testing area in a semi-circle approximately 1.5 m from the entrance. The objects were spaced about 30 cm from each other with five items on the left and five on the right as seen from the central access point.

While Gable and his owner waited behind a barrier, the experimenters placed 10 objects in the testing area. The owner was seated on a chair with Gable on a mat in front of her. Next, the owner asked Gable to bring one target item from this set. All target items were only selected once, and the total number of times an item appeared in a set was balanced across all sets. Target locations were equally distributed between left and right from the central access point. Gable's owner asked Gable to retrieve an item by mentioning the name of the object, and then giving Gable a fetch command with the name of the object: for example, *x; get x*. While Gable searched for the requested item, he could not see his owner or the experimenters, who were located behind two other barriers. Selected items were brought back and dropped in a container next to the owner by Gable, after which he was asked to lie down on his mat again until a new trial started. On most occasions Gable received verbal praise after he had lain down, so that any reward was given following lying down rather than immediately after the object retrieval task.

#### B) Results

Gable correctly retrieved a total of 43 out of the 54 objects his owner claimed that he know the name of (binomial test (*p*(Y≥43 | *n* = 54, *p* = .11) <.001). In trials in which Gable did not retrieve the correct object he either selected an incorrect item without hesitation (five out of 11 times), or hesitated, whined and seemed to wait for further instructions (six out of 11 times). Incorrect trials did not share any obvious features. These results show that Gable reliably knew the words for 43 objects.

### Experiment 2: bias in word generalization after brief familiarization

The purpose of this experiment was to replicate LSJ's standard experimental paradigm [12] with Gable. LSJ found that word generalization is based on object shape, but not on object texture or object size in both young children and adults.

#### A) Methods

**Figure 2:**
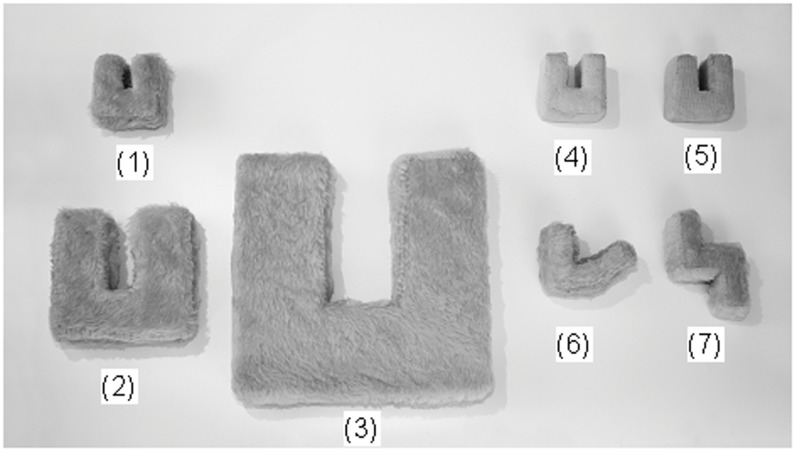
Objects used in experiments 2 and 4. Gable learnt to link the word *dax* with standard object 1: the DAX object (furry light blue 7.6 cm wide). He was asked to select a DAX from pairs of objects including the DAX, size changes 2 (15.2 cm) and 3 (30.4 cm), texture changes 4 (smooth) and 5 (rough), and shape changes 6 and 7.

In a forced choice version of this paradigm LSJ used a new word (for example, *dax*) to name a new object (the standard object in Figure 2), after which participants were presented with pairs of objects similar to those illustrated in Figure 2, and were asked to select the DAX object. We implemented this paradigm with Gable with some essential modifications in relation to the stimuli and the procedures. The standard DAX object was 5.1 cm (two inches) wide in LSJ's experiment. Since such a small size might present a choking hazard for Gable we increased the width of the DAX object to 7.6 cm (three inches), and since nothing is known at present about just noticeable differences in object size discrimination in dogs the three size variations with ratios of 1∶1.25∶4 as used by LSJ were changed to three distinct size variations with ratios 1∶2∶4 in our experiment. Furthermore, in LSJ's experiment the DAX object was made from wood with one texture variation consisting of cloth covered wood and another texture variation consisting of sponge. Since the latter variation might also have presented a choking hazard for Gable our texture variations were based on three different cloth textures covering foam cut-outs. Finally, in LSJ's paradigm children and adults received one exposure of a word-object combination before the experiment in addition to having the DAX object in view during testing. Since the purpose of our experiment was not to investigate Gable's fast-mapping abilities (the ability to remember the mapping of a word to an object by using only one new word-new object pairing; see [1], [4], [10], [11]) Gable was given a 10 minute familiarization of the new object-new name combination before testing. During the 10 minute familiarization Gable received several DAX object-*dax* name pairings in addition to being asked to retrieve the *dax* from among several of his toys for which he knew the names. This familiarization process was congruent with the way in which Gable was normally introduced to a new object-new name combination (see Participant section above and Movie S3). And, since Gable could not be asked to ignore the new object in view when making a choice between two other objects as the human participants had done Gable was reminded of the new object-new name combination before each trial by showing him the object, sounding out the name for it, and allowing him to grasp the object. The trial was then started after a short break during which the DAX object was taken away and two objects were placed in the testing area (see Movie S1).

The objects in Figure 2 were made from foam swimming boards. In order to avoid scent cues every object was covered with the same two layers of cloth applied with different textures outermost, so that every object was always covered by the same kinds of materials, and the objects and the materials they were made of were always stored together. Objects 4 and 5 respectively had smooth and rough light blue cloth on the outside, whereas the standard DAX object and all other objects had furry cloth on the outside with the same light blue color. Objects 2 and 3 were respectively 15.2 cm and 30.4 cm in width, whereas the standard DAX object, as well as all other variants, was 7.6 cm in width.

Gable was allowed to briefly play with all of the objects in Figure 2 (taking them in his mouth 2–3 times) before testing, to avoid him using scent cues left on the new objects by his saliva. In order to avoid scent cues, the objects were also handled by one experimenter only. During the familiarization phase the owner paired the word *dax* with the DAX object by asking Gable to fetch the DAX object by using the *get dax* command several times, after which the DAX was put into several sets of objects for which Gable knew the names, and asking him to fetch the DAX. After the familiarization phase we proceeded with the testing.

It is logically possible to construct 18 unique pairs of two objects out of the seven objects portrayed in Figure 2. Gable was presented with 16 out of those 18 possible pairs; two pairs were randomly selected not to be included in case the testing procedure did not work, and another way of presenting the object pairs needed to be tried out independently. The 16 object pairs were presented in four blocks of four object pairs each. Across all blocks the object pairs were presented in a pseudo-random order, with no more than two objects of the same type on the same side in sequence. In all trials in which the DAX was paired with another object, there were an equal number of trials in which the DAX was on the right and on the left from the point of entry in the testing area.

Gable was tested in the same testing area as described for experiment 1, with the two test items spaced approximately 30 cm from each other on the left and on the right from the central access point. The procedure for asking Gable to retrieve a DAX was identical to that described for experiment 1.

#### B) Results

Gable linked the word *dax* to DAX-sized objects in ten out of ten cases in which he was given the choice between a DAX-sized object and a larger object (binomial test (*p*(Y≥10 | *n* = 10, *p* = .5) <.001), thus confirming that Gable generalized the meaning of the word *dax* to other objects that were of the same size as the DAX object, irrespective of their shape or texture. In those cases in which Gable was able to make a choice between DAX-textured objects and objects with a different texture, and DAX-shaped objects and objects with a different shape he did not appear to have a preference for objects that had the same texture as the standard DAX object (binomial test (*p*(Y≥2 | *n* = 8, *p* = .5) = .86), or objects that had the same shape as the DAX object (binomial test (*p*(Y≥1 | *n* = 8, *p* = .5) = .996) as is the case in humans [12]. As was the case with two and three year old children [12] Gable did not reliably identify a DAX object in relation to the other experimental objects (binomial test (*p*(Y≥3 | *n* = 6, *p* = .5) = .66).

### Experiment 3: investigating Gable's size bias after brief familiarization

The purpose of this experiment was to determine whether the size bias found in experiment 2 was based on Gable preferring the smallest object (for example, because smaller objects are easier to pick up) or whether the size bias was based on a preference for generalizing word meaning to objects of the same size as the standard object.

#### A) Methods

**Figure 3:**
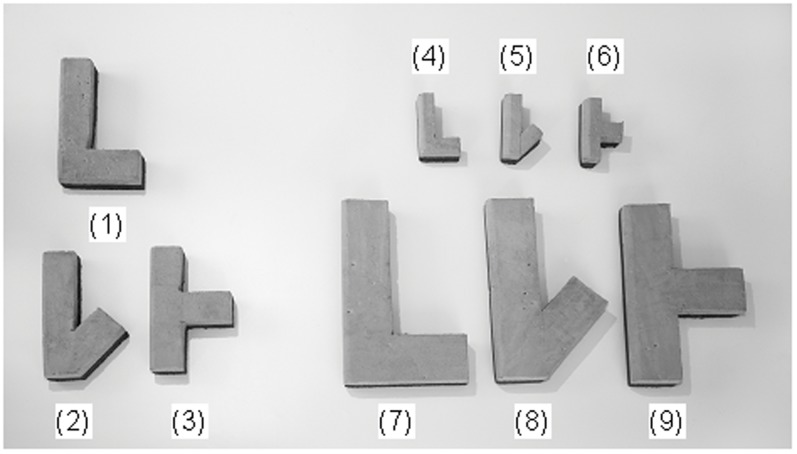
Objects used in experiment 3. Gable learnt to link the word *gnark* with standard object 1: the GNARK object (16 cm * 6 cm). He was asked to select a GNARK from pairs of objects including the GNARK, shape changes 2 and 3, and size changes 4–6 (8 cm * 3 cm) and 7–9 (24 cm * 9 cm).

In experiment 3, object size was pitted against object shape only; thus contrasting a shape bias as found in humans [12] with a size bias as found for Gable in the previous experiment. As in experiment 2 a new word (*gnark*) was used to name a new object (the standard object in Figure 3). Gable was then presented with pairs of objects from those shown in Figure 3, and was asked to select the GNARK object (see Movie S2).

The objects in Figure 3 were again made from foam swimming boards, but not covered with cloth, since texture features were not tested for. The height and width of objects 1, 2 and 3 were 16 cm and 6 cm, for objects 4, 5 and 6 they were 8 cm and 3 cm, and for objects 7, 8 and 9 they were 24 cm and 9 cm.

As in the previous experiment Gable was allowed to play with all of the objects before the experiment to avoid him using scent cues during the experiment, and after an interval, Gable was familiarized with the *gnark* word-GNARK object combination for 10 minutes using the same procedures as in the previous experiment.

The nine objects in Figure 3 lead to 36 logically possible object pairs that could be presented to Gable. Gable was presented with all of these combinations in two sessions of 18 object pairs on two separate days. The object pairs in a session were presented in four different blocks, consisting of two sequences of a set of five trials plus a set of four trials, with breaks in-between sets of trials. Across all blocks the object pairs were presented in a semi-random order, with an equal number of trials in which the GNARK featured on the right side and on the left side from the point of entry in the testing area.

Gable was tested in the same testing area as described before, with the two test items spaced approximately 30 cm from each other on the left and on the right from the central access point. The procedure for asking Gable to retrieve a GNARK was identical to that described for experiment 2.

#### B) Results

Gable demonstrated a word generalization bias for objects with the same size as the GNARK (binomial test (*p*(Y≥13 | *n* = 18, *p* = .5) = .048), but not for objects with a GNARK shape (binomial test (*p*(Y≥12 | *n* = 19, *p* = .5) = .18), despite an overall right side bias (binomial test (*p*(Y≥26 | *n* = 36, *p* = .5) = .006). The right side bias – due to object selection on the right hand side in the final 15 trials in this experiment – could not be attributed to any object features.

Gable correctly identified the GNARK in relation to the other experimental stimuli (binomial test (*p*(Y≥7 | *n* = 8, *p* = .5) = .035, with (*p*(Y≥5 | *n* = 5, *p* = .5) = .03 for the first session in which no right side bias was present), demonstrating that he was able to differentiate between the GNARK and the other objects he was tested with. Finally, in all of those cases where Gable did not choose the medium sized GNARK, he did not prefer the smaller out of two objects (binomial test (*p*(Y≥11 | *n* = 27, *p* = .5) = .88). The latter result, together with a bias for selecting medium sized objects, shows that Gable's bias for selecting the smallest objects in experiment 2 is unlikely to have been due to a general preference for small objects. This experiment thus confirms a size bias in word generalization after a brief new word-new object familiarization period, as well as the absence of a shape bias as found in humans [12].

### Experiment 4: bias in word generalization after extended familiarization

The purpose of this experiment was to repeat the DAX experiment described above, the only difference being a longer exposure for Gable to the *dax* word-DAX object combination. In humans, the shape bias is constant in word generalization; it does not, for example, change to a different kind of bias with age or training [12], [18]. We wanted to find out if this was also the case for Gable.

#### A) Methods

Four months after experiment 2, Gable took the DAX home for 39 days, and was taught to link the word *dax* with the DAX object on a daily basis in different contexts afforded by the home environment and by inserting the DAX into many different sets of toys, and asking Gable to *get the dax*.

The testing in this experiment was identical to the previous DAX experiment, with only one exception: all 18 possible object combinations from Figure 2 were tested, with the exact same stimulus pairs as in the previous DAX experiment, although with an added block of two trials that were not tested before.

#### B) Results

When Gable was familiarized with the *dax* word-DAX object combination for 39 days he selected the DAX in 6 out of 6 trials from among 9 or 10 of his other toys (*p*(Y≥6 | *n* = 6, *p* = .11) = .001), showing that reference for the word *dax* was well established over the 39 day period in relation to his toys. Gable tended to generalize his word knowledge to objects of the same texture (*p*(Y≥8 | *n* = 10, *p* = .5) = .055), but not to objects of the same size (*p*(Y≥3 | *n* = 12, *p* = .5) = .98) or shape (*p*(Y≥5 | *n* = 10, *p* = .5) = .62). As in experiment 2 and in agreement with two and three year old children [12], Gable did not show that he could reliably distinguish between a DAX and other artificial objects that only differed in size, texture or shape (*p*(Y≥4 | *n* = 6, *p* = .5) = .35). These results show that after a long *dax* word-DAX object familiarization period, word generalization was qualitatively different for Gable compared to a short familiarization period, but also compared to word generalization in humans.

### Establishing a possible feature bias in Gable's past input

It is possible that the objects Gable had learned the names for in the three years previous to our experiments were the cause for his bias in word generalization. Feature dissimilarity between the objects he was presented with would in such a case be a key scaffold for word learning. To illustrate: if none of the objects were dissimilar in shape, Gable would not have been able to use shape as a reliable feature for word reference during word learning, but if all objects were dissimilar in shape this would be an ideal feature to scaffold word learning. We therefore investigated possible dissimilarities in shape, size, texture and color of the objects Gable knew the names for.

For each object out of the set of 43 objects for which Gable had demonstrated to know the name, two experimenters independently determined feature dissimilarity in shape, size, texture or color in relation to any other object in the set, and determined feature dissimilarity for a conjunction of two features (for example, shape and size). All objects were examined visually and by touch. Binomial probability tests (against a test proportion of 0.5) established that there were no significant differences between the two experimenters in their evaluations of feature differences or feature conjunction differences, and binomial probability tests (against a test proportion of 0.5) also established that there were no significant differences between the shape, size, texture or color features or pairwise combinations of these features within the set of objects for which Gable knew the words. These results suggest that Gable's bias in word generalization cannot be explained purely by the nature of past input features in word learning.

## Discussion

We have shown that word generalization and the developmental path for acquiring words is qualitatively different in Gable compared to humans. Unlike humans, Gable did not rely on object shape in word generalization. And, although for humans object shape is a stable property in both word comprehension and production [12], [24], Gable's reliance on object size was replaced by a tendency to rely on object texture when he was familiar with a word-object mapping for a longer period of time. Gable's word generalization and word knowledge development are thus qualitatively different from those found in humans, suggesting that the human phenotype in relation to word comprehension may be distinctive compared to the domestic dog. More tests employing the same method to investigate word generalization would be necessary to confirm this idea in relation to the dog as a species.

Why do object names refer to object shape for humans and to object size or even texture for Gable? In order to answer this question it is necessary to establish that objects can only be named if they are categorized or identified. Although touch can give humans reliable information about object identity [27], we primarily rely on vision for object identification [28], [29]. Visual object shape is the most important feature which makes categorization or identification of solid objects possible [28]–[31], and our cognitive system therefore relies mostly on shape for object naming [12]–[24]. The evolutionary history of our sensory systems – with vision taking priority over other sensory systems – seems to have primed humans to take into account visual object shape in object naming tasks.

Why did Gable rely on object size and texture in word generalization in our experiments? Although our analysis of Gable's past input for word learning did not identify a bias in exposure to shape, size, texture or color or binary conjunctions of these features during word learning, it is not entirely possible to rule out a human based bias in the scoring of these features. For example, although we measured the size of the objects along the dimension we deemed relevant for Gable for picking up the object our choice for the relevant dimension could not be independently verified. It is thus possible that in the absence of clear scent cues object size and object texture were important for Gable because these cues were available to him from manipulating the objects with his mouth (bite size and felt texture), or because these cues were part of his past input during word learning as deemed relevant to him. More research is necessary to not only determine the generalizability of the relative importance of object size and object texture discrimination in the dog, but also to determine the relative contribution to word learning and word generalization of scent cues in relation to the object features studied here.

## Supporting Information

Movie S1 **DAX trials.** This clip shows two trials in which Gable was asked to retrieve the DAX (as in experiments 2 and 4). In the break between two trials Gable is reminded of the name for the DAX object.
(MP4)

Movie S2 **GNARK trials.** This clip shows two trials in which Gable was asked to retrieve the GNARK (as in experiment 3). In the break between two trials Gable is reminded of the name for the GNARK object.
(MP4)

Movie S3 **familiarization.** This clip shows Gable being familiarized with the *dax* name for the standard DAX object.
(MP4)
